# IL-6 production in ovarian carcinoma is associated with histiotype and biological characteristics of the tumour and influences local immunity

**DOI:** 10.1054/bjoc.1999.0973

**Published:** 2000-01-18

**Authors:** I Kryczek, M Gryboś, L Karabon, A Klimczak, A Lange

**Affiliations:** Institute of Immunology and Experimental Therapy, Polish Academy of Sciences, 12 Weigl str 53–114, Wroclaw, Poland; 1st Clinic and Department of Gynaecology, Medical Academy, Wroclaw, Poland

**Keywords:** ovarian carcinoma, IL-6, Ki-67, p53, carcinomatous effusion, phenotype

## Abstract

The presence of interleukin (IL)-6 in peritoneal carcinomatous fluid (PCF) and its effect on immune cells composition in PCF in patients with advanced ovarian carcinoma was studied. In 21 out of 30 ovarian carcinoma patients, PCF IL-6 levels were found to exceed those seen in PCFs of patients with gastrointestinal cancer. IL-6 activity was higher in serous/mucinous than in endometrioid and undifferentiated ovarian carcinoma PCF (*P* = 0.05). Ovarian carcinoma PCF IL-6 activities were correlated with serum C-reactive protein levels (*r* = 0.65, *P* = 0.0000, *n* = 25). Ovarian carcinoma PCF leucocyte profile differed from that in blood with respect to: (i) lower percentage of NK and CD8^+^and (ii) higher percentage of B and CD45RO^+^, CD14^+^and HLA-DR^+^cells. The proportions of CD45RO^+^in blood were correlated with IL-6 levels in PCF. Corresponding to PCF ovarian carcinoma tumours were stained for the presence of Ki-67 antigen and p53. The highest proportions of Ki-67^+^cells and cells showing accumulation of p53 were seen in undifferentiated tumours. A low grade of p53 staining was seen in tumours associated with high IL-6 levels in PCF. It was evident that IL-6 production (i) depended on the histiotype of the tumour, (ii) influenced the local immune system in favour of accumulation of B, and T memory cells, and (iii) was higher in patients lacking p53 accumulation. © 2000 CancerResearch Campaign


British Journal of Cancer (2000) **82**(3), 621–628
© 2000 Cancer Research Campaign
DOI: 10.1054/ bjoc.1999.0973, available online at http://www.idealibrary.com on IDEAL®

### IL-6 production in ovarian carcinoma is associated with histiotype and biological characteristics of the tumour and influences local immunity

**I Kryczek[1], M Gryboś[2], L Karabon[1], A Klimczak[1] and A Lange[1]**

[1]Institute of Immunology and Experimental Therapy, Polish Academy of Sciences, 12 Weigl str 53–114, Wrocław, Poland[2] 1st Clinic and Department of Gynaecology, Medical Academy, Wrocław, Poland

**Summary** The presence of interleukin (IL)-6 in peritoneal carcinomatous fluid (PCF) and its effect on immune cells composition in PCF in patients with advanced ovarian carcinoma was studied. In 21 out of 30 ovarian carcinoma patients, PCF IL-6 levels were found to exceed those seen in PCFs of patients with gastrointestinal cancer. IL-6 activity was higher in serous/mucinous than in endometrioid and undifferentiated ovarian carcinoma PCF ($P = 0.05$). Ovarian carcinoma PCF IL-6 activities were correlated with serum C-reactive protein levels ($r = 0.65$, $P = 0.0000$, $n = 25$). Ovarian carcinoma PCF leucocyte profile differed from that in blood with respect to: (i) lower percentage of NK and $CD8^+$ and (ii) higher percentage of B and $CD45RO^+$, $CD14^+$ and $HLA\text{-}DR^+$ cells. The proportions of $CD45RO^+$ in blood were correlated with IL-6 levels in PCF. Corresponding to PCF ovarian carcinoma tumours were stained for the presence of Ki-67 antigen and p53. The highest proportions of $Ki\text{-}67^+$ cells and cells showing accumulation of p53 were seen in undifferentiated tumours. A low grade of p53 staining was seen in tumours associated with high IL-6 levels in PCF. It was evident that IL-6 production (i) depended on the histiotype of the tumour, (ii) influenced the local immune system in favour of accumulation of B, and T memory cells, and (iii) was higher in patients lacking p53 accumulation. © 2000 Cancer Research Campaign

**Keywords:** ovarian carcinoma; IL-6; Ki-67; p53; carcinomatous effusion; phenotype

Ovarian carcinoma is generally resectable and chemotherapy sensitive (Fennelley et al, 1995). In spite of these favourable characteristics the overall cure rate for advanced stage III and IV patients is approximately 20% (Oncolink, http://oncolink.com). This is due to relapses as a result of expansion of chemoresistant clones. Local immunotherapy is a candidate for an adjuvant treatment (Willemse et al, 1990; Giannios et al, 1997). This option is advocated by the local spread of the cancer in the majority of cases and known cytokine generation potential of ovarian carcinoma (Berek et al, 1991; Plante et al, 1994). Interteukin (IL)-6 is a hallmark cytokine of ovarian carcinoma (Berek et al, 1991; Plante et al, 1994). However, it is not known whether: (i) IL-6 production is associated with a specific histiotype of ovarian carcinoma, (ii) IL-6 modifies local immunity and (iii) is associated with biological features of ovarian carcinoma affecting the progression of the disease. The present paper was aimed at addressing all of these questions. It appeared that a high degree of IL-6 activity in peritoneal carcinomatous fluid (PCF) was seen predominately in mucinous/serous cancers which lacked an accumulation of p53 and this cytokine exerted biological impact not only as a factor inducing C-reactive protein (CRP) but also encouraging influx/expansion of memory T- and B-cells.

### PATIENTS AND METHODS

Thirty-five ovarian carcinoma patients (FIGO > IIB), five with ovarian benign cyst and five with ascites in the course of gastrointestinal carcinoma were investigated. The patients did not receive any treatment before the study. FIGO staging in ovarian carcinoma patients was performed at surgery. Histopathology and grading was performed according to the standard procedure using paraffin-embedded tissue sections. Twenty-six patients were eligible for surgical cytoreduction; in others laparotomy revealed neoplastic mass considered unresectable (Table 1).

*Received 3 September 1998*
*Revised 10 June 1999*
*Accepted 3 August 1999*

*Correspondence to*: A Lange

### Cell isolation

PCF was aseptically obtained during surgery either from peritoneal cavity (24 patients) or from ovarian cysts (five patients) or both (six patients). Volume of PCF ranged from 250 to 20 000 ml and the cellularity from $1 \times 10^7$ to $1.2 \times 10^8$ cells $ml^{-1}$ (mean $2.3 \times 10^8$ cells $ml^{-1}$). Volume (and cellularity) of cysts PCF ranged from 150 to $3000 \times 10^6$ ($1–8 \times 10^6$ cells $ml^{-1}$).

Benign cysts fluid volume ranged from 100 to 5000 ml and $0.2–2 \times 10^6$ cells $ml^{-1}$. Both, benign and carcinomatous fluids, were centrifuged (250 ***g***, 10 min at 4°C) to obtain cells and supernatants for further study.

Peripheral blood was collected into lithium-heparin, EDTA and serum collection tubes at the day of surgery for mononuclear cell phenotype, RNA study and protein level measurements, respectively.

### Cell purification

PCF cellular pellet was resuspended in RPMI-1640 ($5 \times 10^6$ cells $ml^{-1}$) and overlaid on two-step density gradient consisting of 100% (density 1.077 g $ml^{-1}$) and 75% (1.057 g $ml^{-1}$) of Ficoll-Metrizoate solution (Gradisol-L, Polfa, Kutno, Poland) and centrifuged

**Table 1** Patient characteristics

| | | |
|---|---|---|
| *Number of patients* | | 35 |
| *Mean age* | | 55 (32–84) |
| Histological type | Serous | 14 (40%) |
| | Mucinous | 4 (11%) |
| | Endometrioid | 6 (19%) |
| | Undifferentiated | 11 (30%) |
| Stage (FIGO): | IIB | 2 |
| | IIC | 2 |
| | IIIA | 3 |
| | IIIB | 2 |
| | IIIC | 19 |
| | IV | 7 |
| *Ovarian carcinoma* | | |
| Surgery | Radical | 12 |
| | Cytoreduction | 14 |
| | Unable | 9 |
| Residual tumour: | <5 cm | 15 |
| | >5 cm | 20 |
| Material | Peritoneal neoplastic effusion | 27 |
| | Peritoneal neoplastic effusion and cyst fluid | 4 |
| | Cyst fluid | 4 |
| *Gastrointestial cancer* | | |
| Number of patients | | 5 |
| Mean age | | 51 (43–62) |
| Residual tumour: | >5 cm | 5 |
| Material | Peritoneal neoplastic effusion | 5 |
| *Benign cyst* | | |
| Mean age | | 47 (34–58) |
| Material | Benign cysts fluid | 5 |

(400 ***g***, 30 min at 18°C) to separate leucocyte mononuclear cells (MNC) and enriched with cancer cell fractions (Freedman et al, 1994). In five cases, cytokeratin 10 and HLA DR antigen (non-polymorphic region) staining was performed in both upper (enriched in cancer cells) and lower (PCF MNC) cell fractions. This staining revealed that the upper fraction was from 50 to 96% keratin-positive cells and from 5 to 30% HLA-DR$^+$ cytokeratin–mesothelial-like cells (Figure 2). Cytofluorograph analysis of lower interphase cells revealed that this fraction was composed of lymphocytes: CD45$^{bright+}$/CD14$^-$ [59.4% + 17.26 s.e.m., $n$ = 16], granulocytes: CD45$^{dimm+}$/CD14$^-$ [15.89% + 9.22 s.e.m., $n$ = 16] and monocytes/macrophages: CD45$^+$/CD14$^+$ [24.7% + 11.6 s.e.m., $n$ = 16]. Peripheral blood mononuclear cells (PBMC) were isolated by standard one-step density gradient centrifugation (1.077 g ml$^{-1}$). All cell fractions (viability > 95% assessed by trypan blue exclusion) were washed, resuspended in RPMI and adjusted to the desired concentration.

### Flow cytometry

One times 10$^6$ cells ml$^{-1}$ of PCF MNC and PBMC were stained with fluorescein isothiocyanate (FITC) or phycoerythrin (PE)-conjugated monoclonal antibodies (mAb) specific for CD3, CD4, CD8, CD16, CD56, CD57, CD22, CD45RO, CD25 and HLA-DR (Becton-Dickinson), read in the Becton-Dickinson FACStar+ instrument and analysed with the use of PC-Lysis software. CD45 and CD14 mAb staining was usually used for leucocyte and lymphocyte gating. CD14 was assessed in leucocyte gate, CD45RO and HLA-DR in both leucocyte and lymphocyte gate, T, B and NK cell markers staining was read in lymphocyte gate.

### Serum and PCF protein measurements

Immunoglobulins (A, G and M), albumin and CRP levels were according to the manufacturer's instruction determined by the use of a NBehring Laser nephelometer.

### Cell lines

7TD1 was a gift from Prof. Van Snik, Ludwig Institute of Cancer Research, Bruxelles; P388 and WEHI 164 were obtained from the Institute of Immunology and Experimental Therapy Polish Academy of Sciences, Wrocław, Poland.

### Assay for IL-6 biological activity

IL-6 level in PCF and serum were measured with the use of 7TD1 hybridoma cell line bioassay (Papadopoulos et al, 1995). 7TD1 cells at the concentration of $2.5 \times 10^6$ cells well$^{-1}$ were plated in 96-well flat-bottomed plates. On day 2 of culture, serial dilutions of experimental samples and standard supernatants and on day 4, 30 µl of 3-(4,5-dimethylthiazal-2-yl)-2,5-diphenyltetrazolium bromide (MTT) were added to each well. Cells were then lysed and collected fluid was read at 540 nm. Results were expressed in units calculated as fraction of a standard sample concentration, which caused 50% of maximal growth as assessed from the curve after probite transformation of the original readings.

Specificity of the bioassay was validated with the use of anti-IL-6 monoclonal antibody (clone BE-8, gift from Dominique Emilie, INSERUM, Clamart, France). Eight randomly chosen PCF samples with known IL-6 activity at the range from 1500 U ml$^{-1}$ to 8000 U ml$^{-1}$ were retested after prior to the bioassay 1 h

© 2000 Cancer Research Campaign incubation at 37°C with neutralizing antibody (5 µg ml$^{-1}$). Neutralization was seen in all tested samples independent on the level of activity and ranged from 90% to 98%.

### Assay for tumour necrosis factor α

Tumour necrosis factor α (TNF-α) level in PCF and serum was measured based on WEHI 164 cell line (Boury et al, 1997). WEHI 164 fibroblast cells at a concentration of $4 \times 10^5$ cells well$^{-1}$ were plated in 96-well, flat-bottomed plates. When the monolayer of WEHI 164 cells became confluent, 0.2 mg ml$^{-1}$ actinomycyne D (Sigma), together with either recombinant TNF-α and experimental samples were added. A 1-day culture (37°C, 5% carbon dioxide) was followed by incubation with 30 µl well$^{-1}$ of MTT. Finally, cells were lysed and samples read at 540 nm and calculated with the use of a probite transformation.

WEHI cell line used in the bioassay was sensitive to recombinant TNF-α (rTNF-α) at concentrations from 0.05 pg ml$^{-1}$ to 700 pg ml$^{-1}$ and this sensitivity was abrogated by TNF-α neutralizing monoclonal antibody (clone: 1825.121, R&D Systems) at concentration 5 µg ml$^{-1}$. TNF-α activity found in two PCF retested samples was neutralized above 90%.

### Immunohistochemical staining of tumour tissue paraffin sections and cytospins

Paraffin block tissue sections were microwave processed (Cattoretti et al, 1992) and stained with the use of the three-step immunoperoxidase technique based on Dako reagents and staining system. Monoclonal, antibody for Ki-67 antigen (MIB1) was kindly provided by J. Gerdes (Forschungs Institut Borstel, Germany) and p53 was purchased from Dako (Clone DO-7). The Ki-67 tumour positivity was graded according to the presence of <10% (+), 10–50% (++) and >50% (+++) of positive cells. p53 expression was evaluated as positive when unequivocal staining was seen and graded as – (no positive cells), + (few cells), ++ (less than 50%) and +++ (majority of cells). All positive p53 tumours showed nuclear staining. Cytospins were stained with the use of the LSAB+ detection kit (Dako) for the presence of cytokeratin (MNF116 mAb against cytokeratin 10, 17, 18, Dako) and HLA-DR (Dako) positive cells.

### Reverse transcriptase polymerase chain reaction

Total RNA was isolated with the use of guanidine isothiocyanate–phenol–chloroform extraction technique. Five microlitres of RNA solution (electrophoretically proved to contain undergradated RNA) was subtracted to reverse transcriptase activity (Stratagene). cDNA used for PCR was standardized against glyceraldehyde 3-phosphate dehydrogenase (GAPDH) mRNA. cDNA was amplified (Biometra UNO II thermocycler) for GAPDH (35 cycles: 95°C: 30 s, 68°C: 30 s and 72°C: 45 s) and for IL-6 (38 cycles: 95°C: 30 s, 60°C: 30 s and 72°C: 45 s) in 25-µl reaction mixture containing cDNA dilution, 0.125 pM dNTP (Gibco-BRL), 1.25 unit of *Taq*-polymerase (Gibco-BRL), polymerase chain reaction (PCR) Tris-HCl buffer (pH 8.3) and 25 pM of 5′ and 3′ sequence primers: (GAPDH: 5′-AACAGCGACACCCACTCCTC-3′, 5′-GGAGGGGAGATTCAGTGTGTT-3′; IL-6: 5′-TAGCCGCCCCACACAGACAG-3′, 5′-GGCTGGCATTTGTTGTTGGG-3′). PCR products (10 µl) were electrophoresed in 1.5% agarose gel and stained with ethidium bromide.

### RESULTS

### IL-6 PCF activity

Mean value +3 standard deviations (3 s.d.) of benign cyst fluid IL-6 activity were considered as a limit of normality. IL-6 activity in all five gastrointestinal cancers PCFs was above this limit. In 67% of ovarian carcinoma patients, PCF IL-6 activity exceeded the mean value +3 s.d. of gastrointestinal cancer PCF IL-6 activity. The values above the latter limit were considered as specific for ovarian carcinoma (Figure 1). IL-6 ovarian carcinoma PCF levels were, in all cases, higher than those found in blood. PCF IL-6 levels and blood levels were merely correlated ($r$ = 0.38, $P$ = 0.076, $n$ = 25).

IL-6 generation was associated with the histiotype of the tumours being higher in serous or mucinous than in endometrioid and undifferentiated tumours (Figure 1).

PCF IL-6 and TNF-α activities were not correlated. The latter inflammatory cytokine was found in all 12 except one ovarian carcinoma PCF and this cytokine activity was correlated with CD14$^+$ cell proportions in PCF MNC ($r$ = 0.88, $P$ = 0.001).

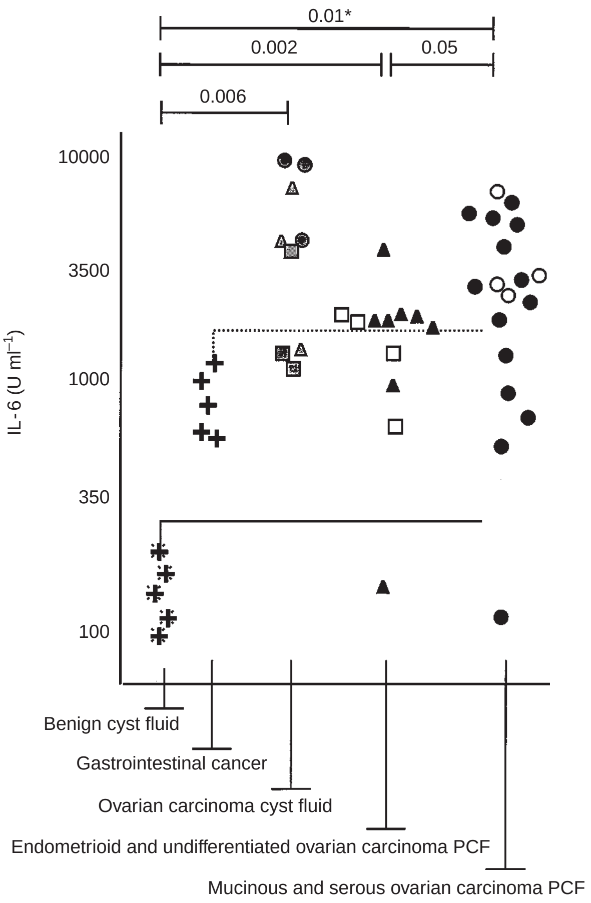

**Figure 1** IL-6 activity (7TD1 bioassay) in benign cyst fluid: BF (*), digestive tract cancer peritoneal fluid: DT cancer (+), and ovarian carcinoma cyst fluid ascites (▲ undifferentiated and ■ endometroid,● serous or ❍ mucous). ------------Mean + 3 × s.d. of gastrointestinal cancer ascites value; ———Mean + 3 × s.d. of benign cyst fluid value

Neutralizing antibody experiments validated the specificity of the 7TD1 and WEHI 164 bioassays used in this study for IL-6 and TNF-α activity measurements in PCF of ovarian cancer patients respectively. In addition WEHI-cell line-based bioassay is not affected by IL-6 and IL-1 (Eskandari et al, 1990), which are the most commonly found cytokines in PCF of ovarian cancer patients. Therefore, we can assume that the results of WEHI bioassay reflected the presence of TNF-α in PCF. To identify more directly cells producing IL-6, PCF cells obtained from ten patients were discriminated into cancer and non-cancer mononuclear cell enriched compartments (see the Methods section and Figure 2) for mRNA study. In all situations IL-6 amplificats were found in reverse transcription PCR (RT-PCR) of PCF cancer fraction populations. In contrast, PCF MNC IL-6 mRNA showed an absence of, or much weaker levels of IL-6 amplificats (Figure 2).

### Biological impact of PCF IL-6

Serum CRP levels were above the normal value range in 96% ovarian carcinoma cases and were correlated with PCF IL-6 values ($r$ = 0.65, $P$ = 0.0000, $n$ = 25). To investigate the local effect of IL-6 production phenotype, profile of PCF MNC was determined and compared to that of blood. PCF MNC contained more $CD14^+$ and B lymphocytes ($CD22^+$ or $CD19^+$ cells) than PBMC. These differences probably contributed to the prevalence of HLA-$DR^+$ cells in PCF (Figure 3). In the T-cell compartment there were more $CD4^+$ than $CD8^+$ cells in PCF MNC as compared to blood which resulted in a higher CD4/CD8 ratio in PCF MNC than in PBMC (4.8 + 0.94 vs 2.6 + 0.4, $P$ = 0.03, Student's $t$-test for paired samples). PCF MNC had a higher proportion of CD45RO+ cells than that found in PBMC but lower of $CD56^+$ and $CD57^+$ cells (Figure 3). To address more directly the effect of IL-6 on cellular profile of PCF MNC the coefficient of correlation between the proportions of cells and the levels of IL-6 was calculated. It appeared that only CD45RO but not all T- (CD3) or B- (CD19 or CD20) cell fractions were positively correlated with the levels of IL-6 in PCF ($r$ = 0.71, $P$ = 0.001, $n$ = 21; Figure 4).

There was no direct association between the proportions of B-cells and IL-6 levels. IL-6 supports B-cell differentiation, and production of IgG is influenced to a larger degree than that of IgA (Kishimoto, 1989). Therefore, IgG to IgA ratio was analysed in PCF and serum. In PCF IgG: IgA was higher than that in blood (3.8 + 0.5 vs 4.55 + 0.55, $P$ = 0.001, Student's $t$-test for paired samples). There was a fair degree correlation between IL-6 ascites level and preoperative platelet count ($r$ = 0.26, $P$ = 0.059).

### Ki-67 and p53 positivity and histiotype of ovarian carcinoma and IL-6 generation potential

Undifferentiated tumours had higher fractions of Ki-$67^+$ cells and $p53^{+++}$ cells (unequivocally nuclear staining) than differentiated adenocarcinomas (Figure 5). p53 extensive staining was inversely correlated with IL-6 generation potential of ovarian carcinoma tumours. The mean value of all PCF IL-6 levels was used as a cut-off point to discriminate cases with tumours having higher (above the mean) and lower IL-6 generation potential. It appeared that tumours with $p53^{+++}$ staining had IL-6 PCF levels more frequently below this cut-off point than tumours with less pronounced p53 staining (10/15 vs 2/10, $P$ = 0.03, Fisher's exact test, Figure 6).

### DISCUSSION

To survive in invaded tissue, carcinoma cells produce factors enabling them accommodation, vascularization, growth and metastasizing potential (Berger et al, 1995; Luca et al, 1997). These substances frequently of cytokine-like characteristics operate in an autocrine or paracrine fashion. IL-6 is known to be a growth factor for some lymphomas (Hsu et al, 1993) and is produced by some solid tumours, e.g. renal (Chang et al, 1997) and ovarian carcinomas (Berek et al, 1991; Plante et al, 1994). IL-6 has pleiotropic activity that includes reactivity with cancer cells (Berger et al, 1995; Luca et al, 1997) and influences some body systems in a distant way (Blay et al, 1997). It has been demonstrated that ovarian carcinoma patients with a high serum IL-6 level had thrombocytosis and anaemia (Gastl et al, 1993). However, little is known whether IL-6 generated locally site influences ovarian carcinoma cells and local immunity.

IL-6 is an immunostimulator enhancing expression of CD54 antigen (Hutchins et al, 1994) which may increase tumour–T-cell interactions. These characteristics of IL-6 promoted some efforts to transfect cancer cells with IL-6 gene to increase immunogenic potential of carcinoma cells (Cao et al, 1996). Each manipulation with a genome may in addition to introducing a given gene alter the biology of transfected cell(s) thus the final effect of transfection depends on the function of the introduced gene and on the non-specific alteration of cancer cells genome (Runnebaum, 1997). Therefore, tumours constitutively producing IL-6 can serve as a model to study the influence of IL-6 on local and systemic immune function in cancer patients.

In this study we analysed 35 patients with ovarian carcinoma. Ovarian cells normally produce IL-6 and this ability may persist in cancer cells originated from ovarian granulosa cells (Machelon et al, 1997). It has been previously reported that papillary ovarian carcinoma are greater producers of IL-6 than non-papillary ones (Kutteh, 1992) but the presence of association between the histiotype and IL-6 production has not been so far shown. The present study substantiated that IL-6 at significant concentration was seen in PCF of serous-mucinous but not of less differentiated and endometrioid carcinomas (Figure 1). This is a novel finding. The presence of the association between the histiotype of ovarian carcinoma and IL-6 levels also shows that IL-6 level depends on the tumour histology but not at the stage or invasiveness of the neoplastic process. Patients in all histiotype tumour categories did not differ with respect to stage (Table 1). Similarly, carcinoma ascites contained much less IL-6 than PCF of the majority of mucinous/serous cancer patients (Figure 1). The ovarian carcinoma cells origin of IL-6 was further indicated by the presence of IL-6 mRNA in cell fraction enriched with carcinoma cells rather than in PCF MNC fraction (Figure 2).

Mesothelial cells may also produce IL-6 (Offner et al, 1995) and these cells were present in PCF cancer fractions (Figure 2). The contribution of mesothelial cells to the ascites IL-6 should be similar in ovarian carcinoma of different histiotype and in gastrointestinal cancer. PFC cells profile of ovarian carcinoma patients characterized with: (i) lower fraction of NK cells; this represented more common phenomenon of depletion of cytotoxic cells in cancer environment (Karimine et al, 1994; Matsuda et al, 1995), (ii) lower fraction of $CD8^+$ $CD3^+$ cells, this has also been described in tumour infiltrating lymphocyte (TIL) population in gynaecological cancers (Schondorf et al, 1997) and some colon

 *© 2000 Cancer Research Campaign*

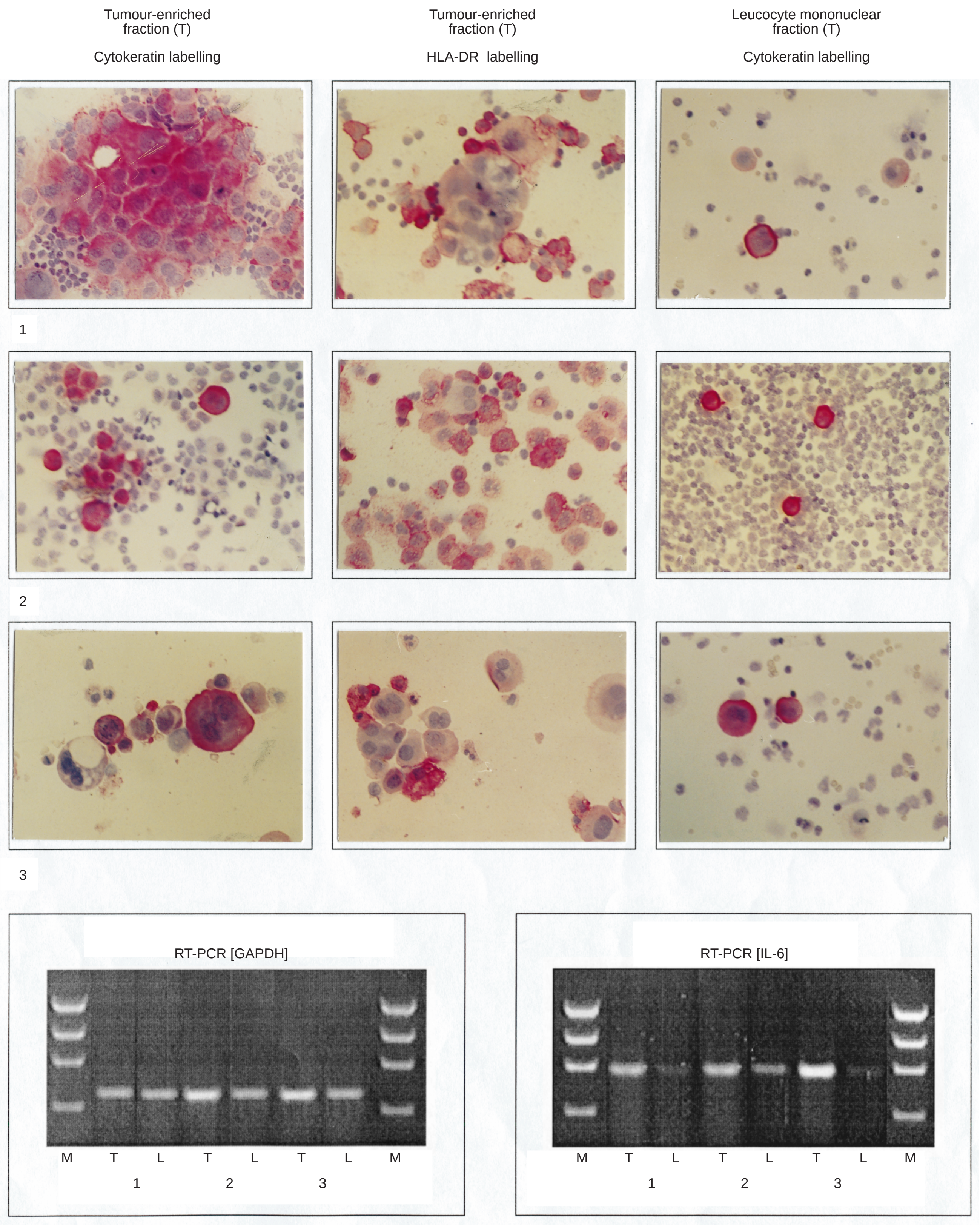

**Figure 2** Cytokeratin and HLA-DR positive cells in PCF MNC and tumour enriched fractions with respective content of GAPDH and IL-6 mRNA (numbers reflect different patients tested). Unstained cells represent mesothelial, cancer cells and leucocytes in (i) cytokeratin and (ii) HLA-DR labelled tumour enriched fraction and (iii) cytokeratin labelled PCF MNC fraction, respectively.

© 2000 Cancer Research Campaign

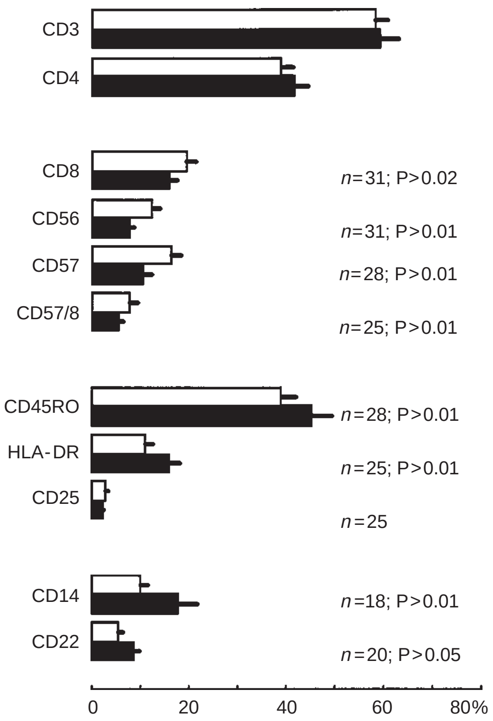

**Figure 3** Percentage of mononuclear cell population (mean ±SEM) in peripheral blood ( ) and PCF ( ) of ovarian carcinoma patients. ● T-test for paired samples.

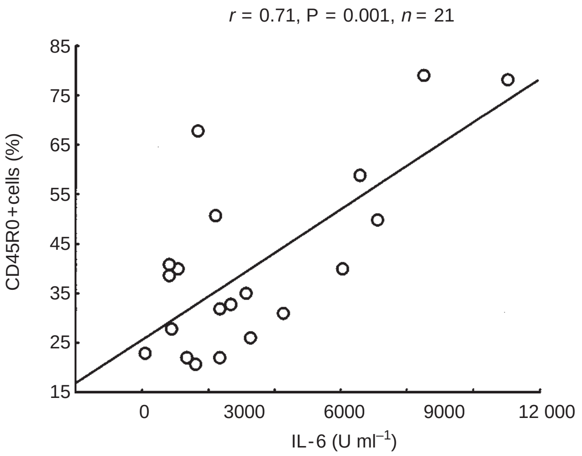

**Figure 4** Correlation between percentage of CD45RO+ cells and IL-6 activity in PCF of ovarian carcinoma patients. *P*=0.001 *t*-test for paired samples n=13

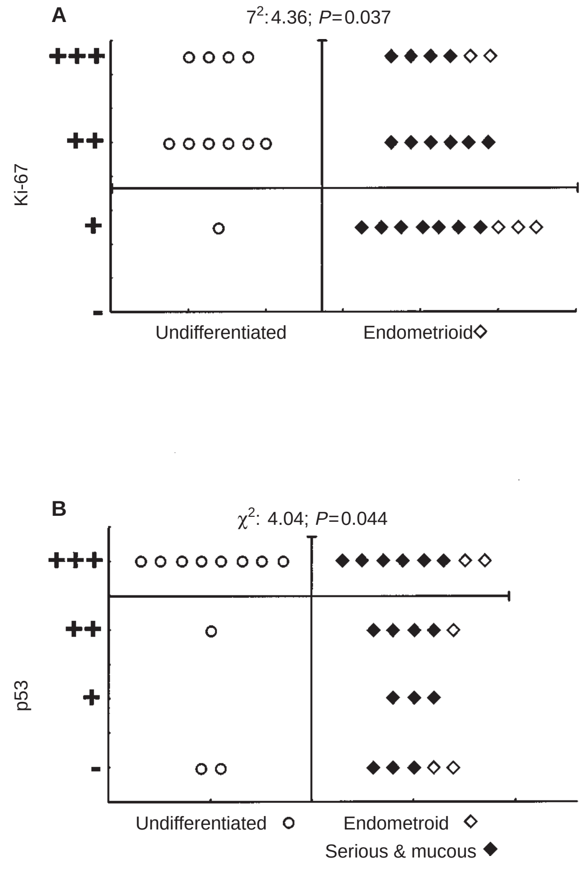

**Figure 5** Accumulation of p53 (**A**) and expression of Ki-67 antigen (**B**) in ovarian carcinoma tumours carcinomas (Wimmenauer, 1994), (iii) an excess of CD19$^+$/CD20$^+$ and (iv) the higher fraction of CD45RO$^+$ cells (Figure 3). The latter two differences have not so far been defined and can be attributed to the presence of IL-6 in ascites. The highest proportions of CD19$^+$/CD20$^+$ cells were seen in ascites of serous/mucinous tumours that identified with a high IL-6 production (data not shown). The proportion of CD45RO$^+$ cells was directly correlated with the PCF IL-6 levels (Figure 4). IL-6 influenced the local immunity favouring B and T memory cells accumulation. These differences might have their functional impact. Ovarian carcinoma PCF immunoglobulins were preferably enriched in IgG. IL-6 injected intraperitoneally is able to augment the secondary response to SRBC in mice more than tenfold (Takatsuki et al, 1988). Whether IL-6-dependent accumulation of CD45RO$^+$ cells reflected more refined immune system recognition of ovarian carcinoma cells has still to be documented. However, it has been illustrated that IL-6 in concert with TNF-α and IL-2 promotes expansion of CD45RO$^+$ cells even in the absence of antigen stimulation (Unutmaz et al, 1994). In PCF TNF-α activity was found in all 16 examined cases. IL-2 mRNA was seen in two out of three samples studied so far (not shown).

The key question is whether IL-6 promotes ovarian carcinoma cell proliferation. This was not the case because there was no association between the proportion of Ki-67$^+$ cells and IL-6 activity (Figure 5). However, p53 was less frequently accumulated in IL-6 producing tumours (Figure 6). The origin of this association, which has not so far been reported, cannot be concluded from the present study. This could be an indirect reflection of an association between serous/mucinous histiotype with a high potential to produce IL-6 (Figure 1) and a low degree of p53 accumulation (Figure 6). However, an association between accumulation of p53 and local TNF-α activity has already been reported (Gotlieb et al, 1994). IL-6 was found to induce apoptosis associated with the activation of wild-type p53 in myeloid leukaemic cells (Yonish-Rouach et al, 1991). Therefore, the local cytokine environment may influence the susceptibility of cancer cells to the damage of genome. In a recent published paper, Asschert et al (Asschert et al,

 © 2000 Cancer Research Campaign

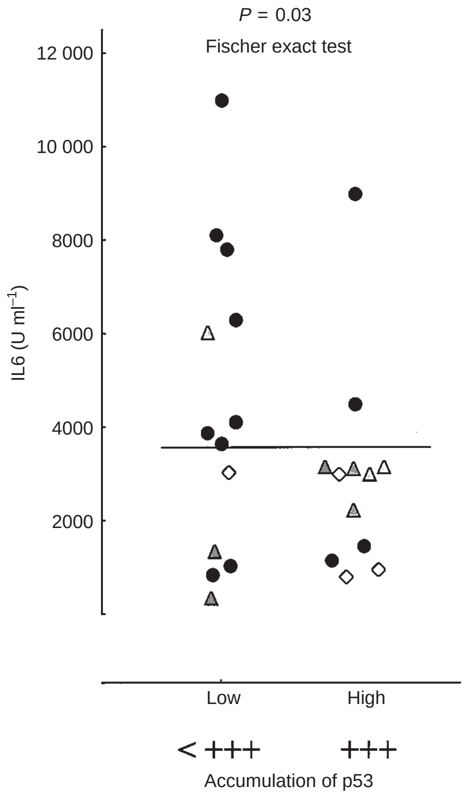

**Figure 6** IL-6 activity in ascites and p53 accumulation in patients with (●) serous or mucous, (▲) undifferentiated and (◆) endometroid OC. ——— Mean value of the whole group

1997) suggested that p53-positive tumours frequently express cytokines. In the latter paper the Northern blot instead of a bioassay or more sensitive RT-PCR was employed. The number of cases was rather limited and the association was valid for macrophage colony-stimulating factor but not IL-6 mRNA.

Data presented in this paper showed that patients characterized either by high IL-6 production or by p53 accumulation prevailed in the ovarian cancer group (18 out of 25, Figure 6). In the normal epithelial cells IL-6 was found to increase bcl-2 expression preventing apoptosis (Rollwagen et al, 1998). Bcl-2 mRNA is present in a proportion of ovarian cancer tumours (Diebold et al, 1996; Herod et al, 1996 and own unpublished observation) and was shown to be independent from p53 prognostic factor (Diebold et al, 1996; Herod et al, 1996). Found in this study the inverse correlation between p53 accumulation and IL-6 generation potential (Figure 6) suggests that in ovarian cancer population two independent mechanisms are involved in tumour survival. One is involved with IL-6 production and the other with p53 mutation. Hypothetically ovarian tumours take an advantage of any of these two mechanisms to survive. Alternatively, the reported negative association between p53 accumulation and high IL-6 generation potential may only show that cancer cells with damaged genome, which was reflected by the accumulation of p53, failed to maintain the ability to produce constitutively IL-6.

### ACKNOWLEDGEMENTS

We thank Prof. Sikora for critical comments to our manuscript. This work was supported by the Polish State Committee for Scientific Research (Grant no. 4 PO5B 066 12).

### REFERENCES

Arinaga S, Karimine N, Nanbara S, Inoue H, Nakashima H, Ueo H and Akiyoshi T (1994) Lymphokine-activated killer cell activity of peripheral blood, spleen, regional lymph node, and tumour infiltrating lymphocytes in gastric cancer patients. *J Surg Oncol* **55**: 179–185

Asschert JG, Vellenga E, Hollema H, van der Zee AG and de-Vries EG (1997) Expression of macrophage colony-stimulating factor (M-CSF), interleukin-6, (IL-6), interleukin-1 beta IL-1 beta), interleukin-11 (IL-11) and tumour necrosis factor-alpha (TNF-alpha) in p53-characterised human ovarian carcinomas. *Eur J Cancer* **33**: 2246–2251

Berek JS, Chung C, Kaldi K, Watson JM, Knox RM and Martinez-Maza (1991) O Serum interleukin 6 levels correlate with disease status in patients with epithelial ovarian cancer. *Am J Obstet Gynecol* **164**: 1038–1043

Berger DP, Herbstritt L, Dengler WA, Marme D, Mertelsmann R and Fiebig HH (1995) Vascular endothelial growth factor (VEGF) mRNA expression in human tumor models of different histologies. *Ann Oncol* **6**: 817–825

Blay JY, Rossi JF, Wijdenes J, Menetrier-Caux C, Schemann S, Negrier S, Philip T and Favrot-M (1997) Role of interleukin-6 in the paraneoplastic inflammatory syndrome associated with renal-cell carcinoma. *Int J Cancer* **72(3)**: 424–430

Boury NM, Stabel TJ, Kehrli M Jr and Taylor M (1997) Comparison of the PK(15)- and WEHI 164 (clone 13)-based bioassays for detection of porcine tumor necrosis factor. *Am J Vet Res* **58**: 1115–1119

Cattoretti G, Becker M, Key G, Duchrow M, Schluter C, Galle J and Gerdes J (1992) Monoclonal antibodes against recombinant parts of the Ki-67 antigen (Mib 1 and Mib 3) detect proliferating cells in microwave-processed formalid-fixed paraffin sections. *J Pathol* **168**: 357–363

Cao X, Chen C, Zhang W, Tao Q, Yu Y and Ye T (1996) Enhanced efficacy of combination of IL-2 gene and IL-6 gene-transfected tumor cells in the treatment of established metastatic tumors. *Gene Ther* **3**: 421–426

Chang SG, Lee SJ, Lee SJ, Kimi JI, Jung JC, Kim H and Hoffman RM (1997) Interleukin-6 production in primary histoculture by normal human kidney and renal tumor tissues. *Anticancer Res* **17**: 113–115

Eskandari M, Nguyen D, Kunkel S and Remik D (1990) WEHI 164 subclone 13 assay for TNF: sensitivity, specificity, and reliability. *Immunol Invest* **19**: 69–79

Fennelley D (1995) Dose intensity in advanced ovarian cancer: have we answered the question? *Clin Cancer Res* **1**: 575–582

Freedman RS, Tomasovic B, Templin S, Atkinson EN, Kudelka A, Edwards CL and Platsoucas CD (1994) Large-scale expansion in interleukin-2 of tumor-infiltrating lymphocytes from patients with ovarian carcinoma for adoptive immunonotherapy. *J Immunol Method* **167**: 145–160

Gastl G, Plante M, Finstad CL, Wong GY, Federici MG, Bander NH and Rubin SC (1993) High IL-6 levels in ascitic fluid correlate with reactive thrombocytosis in patients with epithelial ovarian cancer. *Br J Haematol* **83**: 433–441

Giannios J and Ioannidou-Mouzaka L (1997) Molecular aspects of breast and ovarian cancer. *Eur J Gynaecol Oncol* **18**: 387–393

Gotlieb WH, Watson JM, Rezai A, Johnson M, Martinez-Maza O and Berek JS (1994) Cytokine-induced modulation of tumor suppression gene expression in ovarian cancer cells: up-regulation of p53 gene expression and induction of apoptosis by tumor necrosis factor-α. *Am J Obstet Gynecol* **170**: 1121–1130

Hsu SM, Waldron JW JR, Hsu PL and Hough AJ Jr (1993) Cytokines in malignant lymphomas: review and prospective evaluation. *Hum Pathol* **24**: 1040–1057

Hutchins D and Steel C (1994) Regulation of ICAM-1(CD54) expression in human breast cancer cell lines by interleukin 6 and fibroblast-derived factors. *Int J Cancer* **58**: 80–84

Kishimoto T (1989) The biology of IL-6. *Blood* **74**: 1–10

Kutteh C (1992) Quantitation of tumor necrosis factor-alpha, interleukin-1 beta, and interleukin-6 in the effusion of ovarian epithelial neoplasms. *Am J Obstet Gynecol* **167**: 1864–1869

Luca M, Huang S, Gershenwald JE, Singh RK, Reich R and Bar-Eli M (1997) Expression of interleukin-8 by human melanoma cells up-regulates MMP-2 activity and increases tumor growth and metastasis. *Am J Pathol* **151**: 1105–1113

Machelon V, Nome F, Durand-Gasselin I and Emilie D (1997) Tumor necrosis factor-alpha induces interleukin-6 mRNA and protein in human granulosa luteinizing cells via protein tyrosine kinase without involving ceramide. *Mol Cell Endocrinol* **126**: 173–184

Matsuda M, Petersson M, Lenkei R, Taupin JL, Magnusson I, Mellstedt H, Anderson P and Kiessling R (1995) Alterations in the signal-transducing molecules of T cells and NK cells in colorectal tumor-infiltrating, gut mucosal and peripheral lymphocytes: correlation with the stage of the disease. *Int J Cancer* **61**: 765–772

Offner FA, Obrist P, Stadlmann S, Feichtinger H, Klingler P, Herold M, Zwierzina H, Hittmair A, Mikuz G and Abendstein B (1995) IL-6 secretion by human peritoneal mesothelial and ovarian cancer cells. *Cytokine* **7**: 542–547

© 2000 Cancer Research Campaign

Oncolink – NCI/PDQ: Ovarian epithelial cancer – updated 04/97 (http://ocolink.com).
Papadopoulos NG, Dedoussis GV, Baxevanis CN and Papamichail M (1995) Bioassay vs. immunoassay for quantification of interleukin-6 in biological fluids. *J Clin Lab Anal* **9**: 234–237
Plante M, Rubin SC, Wong GY, Federici MG, Finstad GL and Gastl GA (1994) Interleukin-6 level in serum and ascites as a prognostic factor in patients with epithelial ovarian cancer. *Cancer* **73**: 1882–1887
Runnebaum I (1997) Basics of cancer gene therapy. *Anticancer Res* **17**: 2887–2890
Schondorf T, Engel H and Lindemann C (1997) Cellular characteristics of peripheral blood lymphocytes and tumour-infiltrating lymphocytes in patients with gynaecological tumours. *Cancer Immunol Immunother* **44**: 88–96
Takatsuki F, Okano A, Suzuki C, Chieda R, Takahara Y, Hirano T, Kishimoto T, Hamuro J and Akiyama Y (1998) Human recombinant interleukin 6/B cell stimulatory factor 2 (IL-6/BSF-2) augments murine antigen-specific antibody responses. In vitro and in vivo. *J Immunol* **141**: 3072
Unutmaz D, Piler P and Abrignani S (1994) Antigen-independent activation of naive and memory resting T cells by a cytokine combination. *J Exp Med* **180**: 1159–1164
Willemse PH, de Vries EG, Mulder NH, Aalders JG, Bouma J and Sleijfer DT (1990) Intraperitoneal human recombinant interferon alpha 2b in minimal residual ovarian cancer. *Eur J Clin Oncol* **26**: 353–358
Wimmenauer S, Keller H, Rahner S, Kirste G, Von-Bergwelt M, Meyer A, Von-Kleist S and Farthmann EH (1994) Phenotypical and functional characteristics of tumor-infiltrating lymphocytes from colon carcinomas stimulated with rIL-2 and rIL-4 in vitro: comparison with lymphocytes of the normal colon mucosa and the peripheral blood. *Anticancer Res* **14**: 963–968

 *© 2000 Cancer Research Campaign*
